# *Drosophila* DDX3/Belle Exerts Its Function Outside of the Wnt/Wingless Signaling Pathway

**DOI:** 10.1371/journal.pone.0166862

**Published:** 2016-12-28

**Authors:** Fabian H. Jenny, Konrad Basler

**Affiliations:** Institute of Molecular Life Sciences, University of Zurich, Zurich, Switzerland; Simon Fraser University, CANADA

## Abstract

The helicases human DDX3 and *Drosophila* Belle (Bel) are part of a well-defined subfamily of the DEAD-box helicases. Individual subfamily-members perform a myriad of functions in nuclear and cytosolic RNA metabolism. It has also been reported that DDX3X is involved in cell signaling, including IFN-α and IFN-β inducing pathways upon viral infection as well as in Wnt signaling. Here we used a collection of EMS-induced *bel* alleles recovered from a Wingless (Wg) suppressor screen to analyze the role of the *Drosophila* homolog of DDX3 in Wg/Wnt signaling. These EMS alleles, as well as a P-element induced null allele and RNAi-mediated knock down of *bel*, all suppressed the phenotype of ectopic Wg signaling in the eye. However, they did not affect the expression of known Wg target genes like *senseless*, *Distalless* or *wingful/Notum*. Ectopic Wg signaling in eye imaginal discs induces apoptosis by increasing *grim* expression. Mutations in *bel* revert *grim* expression to wild-type levels. Together, these results indicate that Bel does not function as a core component in the Drosophila Wg pathway, and that mutations affecting its helicase function suppress the effects of ectopic Wg signaling downstream of the canonical pathway.

## Introduction

Eukaryotic helicases are divided into several families, of which the DEAD-box helicases constitute the largest [1], characterized by highly conserved sequence motifs and a distinct structure [2]. Common to all DEAD-box proteins are their ATPase and RNA helicase functions [3,4]. The human DDX3 and Drosophila Belle (Bel) helicases form part of a well-defined subfamily of the DEAD-box helicases; their genes even exhibit a shared exon/intron structure indicative of a common ancestor [5]. Individual subfamily-members perform a myriad of functions in nuclear and cytosolic RNA metabolism. One of the two human orthologs of DDX3, DDX3X, has been implicated in transcription, pre-mRNA splicing, nuclear RNA export, and translation and is present in cytoplasmic mRNA granules [5]. It has also been reported that DDX3X is involved in cell signaling, including a role in IFN-α and IFN-β inducing pathways upon viral infection [6] as well as in Wnt/Wg signaling [7]. A mechanism for DDX3’s role in Wnt signaling was proposed by Cruciat et al. (2013) who suggested that DDX3 acts as a regulatory subunit of casein kinase 1 epsilon (CK1-ε), regulating the phosphorylation of Dishevelled (Dsh)[7]. This signaling function was studied in human cell lines and in *Xenopus* as well as in *C*. *elegans* development, and, surprisingly, mapped to regions in the C-terminus of DDX3, which are not part of the helicase domains (amino acids 456–662).

Here we used a collection of EMS-induced *bel* alleles recovered from a Wg suppressor screen (FH Jenny, M Hediger Niessen et al., manuscript in preparation) to analyze the role of the *Drosophila* homolog of DDX3 in Wg signaling. Unexpectedly, our results indicate that Bel exerts its function(s) downstream (and thus outside) of the endogenous Wnt/Wg pathway in Drosophila.

## Materials and Methods

### Drosophila melanogaster strains and genetics

All crosses were done at 25°C. The following fly lines were used in the experiments:

*y*, *w*, *ey-Flp; +; FRT82*, *cl*
*y*, *w*, *ey-Flp; +; FRT82*, *bel[3R*.*596*.*4] / TM6B(Hu*, *Tb)*
*y*, *w*, *ey-Flp; +; FRT82*, *bel[74407] / TM6B(Hu*, *Tb)* [8]
*y*, *w*, *ey-Flp; +; sev>y[+]>wg*, *FRT82*, *bel[74407] / TM6B(Hu*, *Tb)*
*y*, *w*, *ey-Flp; +; sev>y[+]>wg*, *FRT82*, *bel[3R*.*362*.*1] / TM6B(Hu*, *Tb)*
*y*, *w*, *ey-Flp; +; sev>y[+]>wg*, *FRT82*, *bel[3R*.*382*.*1] / TM6B(Hu*, *Tb)*
*y*, *w*, *ey-Flp; +; sev>y[+]>wg*, *FRT82*, *bel[3R*.*447*.*1] / TM6B(Hu*, *Tb)*
*y*, *w*, *ey-Flp; +; sev>y[+]>wg*, *FRT82*, *bel[3R*.*596*.*4] / TM6B(Hu*, *Tb)*
*y*, *w*, *ey-Flp; +; sev>y[+]>wg*, *FRT82*, *bel[3R*.*599*.*8] / TM6B(Hu*, *Tb)*
*y*, *w*, *ey-Flp; +; sev>y[+]>wg*, *FRT82*, *bel[3R*.*632*.*3] / TM6B(Hu*, *Tb)*
*y*, *w*, *ey-Flp; +; sev>y[+]>wg*, *FRT82*, *pyog[S123] / TM6B(Hu*, *Tb)* (based on line 7209 from Bloomington Drosophila Stock Center, donor Mariann Bienz)
*y*, *sc*, *v; +; P{y[+t7*.*7] v[+t1*.*8] = TRiP*.*GL00205}attP2* (*bel* RNAi)
*P{KK100724}VIE-260B* (*pygo* RNAi)

### Immunochemistry in imaginal discs

In some experiments homozygous clones were induced by mitotic recombination. In eye imaginal discs an *ey-Flp* transgene fulfilled this task. In wing imaginal discs a *hs-Flp* construct was used: here we induced the transgene by a heat shock of 45 minutes at 37°C 48 to 72 hours after egg laying.

Eye or wing imaginal discs were dissected from late wandering 3rd instar larvae and dissected/inverted in PBS (phosphate buffered saline). They were collected in a small Eppendorf tube containing PBS. The PBS was removed and a paraformaldehyde fixative (4% paraformaldehyde and 1%Triton in PEM (0.1M Pipes, pH 6.9 / 1mM EGTA, pH 8.0 / 2mM MgSO4)) was applied for 30 minutes. Afterwards the samples were washed three times for 20 minutes at room temperature with PBT (130mM NaCl / 7mMNa2HPOP4 / 3mM KH2PO4 / 0.1% Tween 20 or Triton-X-100, pH 7.2.)/Na-Azide. Primary antibody (or multiple primary antibodies in sequence) were applied to the samples and incubated for two hours at room temperature on a slow shaker (alternatively overnight at 4°C). The following antibodies were used in our experiments:

- Mouse anti-Wg antibody (monoclonal) from DSHB (concentrate) used in a 1:300 dilution.
- Rat anti-Dll antibody (polyclonal) from Steve Cohen's lab used in a 1:500 dilution (consumed and unavailable).
- Guinea pig anti-Sens antibody (polyclonal) from Hugo Bellen's lab used in a 1:300 dilution.
- Rabbit anti-Bel antibody from Paul Lasko's lab used in a 1:500 dilution.

After the treatment with the primary antibody, the samples were briefly washed three times with 1% heat-inactivated goat serum in PBT/Na-Azide and then incubated in this solution for 30 minutes. Afterwards the fluorescent secondary antibody was applied for two hours at room temperature (or over night at 4°C) to the sample:

- Goat anti-guinea pig Alexa 568 antibody from Life Technologies used in a 1:400 dilution.
- Goat anti-rat Alexa 596 antibody from Life Technologies used in a 1:400 dilution.
- Goat anti-mouse Alexa 596 antibody from Life Technologies used in a 1:400 dilution.
- Goat anti-rabbit Alexa 596 antibody from Life Technologies used in a 1:400 dilution.
- Goat anti-mouse Alexa 488 antibody from Life Technologies used in a 1:400 dilution.
- Goat anti-rabbit Alexa 488 antibody from Life Technologies used in a 1:400 dilution.
- Goat anti-rabbit Alexa 405 antibody from Life Technologies used in a 1:400 dilution.

In some experiments 4’,6’-Diamidin-2-phenylindol (DAPI, 1μl/100μl of solvent) was applied together with the secondary antibody to stain the nuclei. After the secondary antibody treatment, the samples were washed three times with PBT/Na-Azide and destained for one hour in PBT/Na-Azide. Discs were mounted on microscopy slides with VectaShield (Vector Laboratories).

For the visualization of cell death, LysoTracker Red (Life Technologies) was used according to the manufacturer’s specifications.

### Chain termination sequencing

A single fly was put in a thin-walled PCR tube and frozen for ca. 20min at -20°C. It was then squashed with a pipette tip containing 40μl of SB-buffer (10mM Tris-HCl (pH 8.2), 1mM EDTA, 25mM NaCl, 0.2% Triton X in ddH2O). 10ul Proteinase K (20mg/ml) was added. DNA was extracted by incubating the sample for 30 minutes at 37°C and Proteinase K was inactivated by incubating the sample for 5 minutes at 95°C. Regions of interest were amplified using polymerase chain reaction (PCR), using GoTaq G2 Hot Start Kit from Promega with default concentrations but downscaled to 20μl reactions. The sequencing was performed on an Applied Biosystems/Hitachi 3730 DNA Analyzer. For the sequencing reaction the BigDye Terminator v3.1 Cycle Sequencing Kit (Applied Biosystems) was used. The primers used for the sequencing of *bel* alleles are listed in Table 1.

**Table 1:** List of primers used for sequencing *bel* alleles.

Primer Name	Primer Sequence
bel_gene_1	CACAAAGTGCAACACCAA
bel_gene_2	ACGAGCGATAGAGACATTA
bel_gene_3	TAAATGCACTGTGTTCCC
bel_gene_4	ACATCTGGTTGAGAATCG
bel_gene_34.1	CAAGACTTCCACTAACTC
bel_gene_34.2	CTTGGTCCTCTAATATGC
bel_gene_34.3	GCAACAGAAGCTACAACA
bel_gene_34.4	GTTGTAGTTGTCCTCGAA
bel_gene_34.5	GCGATTGTTGTAGCTTCT
bel_gene_5	TTATGACAAACCGACACC
bel_gene_6	TCCGAACTCATATCCTCC
bel_gene_56.1	CCAAGAAGTTCGCCTATC
bel_gene_56.2	TGAGGCTGATCGTATGTT
bel_gene_56.3	CATCTGAGAACATTACGC
bel_gene_56.4	CGTTATTAACTTTGACCTGC
bel_gene_56.5	GATAGGCGAACTTCTTGG
bel_gene_56.6	TGTCCAACATACGATCAG
bel_gene_56.7	GCTTGTCGGGTTCATAGA
bel_gene_56.8	CTAGTGACGGGATGATTG
bel_gene_56.9	GACATACTCCTCCACATC
bel_gene_7	GGATGTGGAGGAGTATGT
bel_gene_8	CTCCGTTTGGTTATTTGC
bel_gene_78.1	GATTACCGTCAAAGCTCT
bel_gene_78.2	GCAGAAACTTAACAGTGG
bel_gene_78.3	CTTTGACGGTAATCCCTG
bel_gene_78.4	ACTGATTACTGCTGATGG

Primers 1, 2, 3, 4, 5, 6, 7 and 8 were used for PCR amplification and re-sequencing. All primers with double digits were used for sequencing the amplicon between primers 3 and 4 (34.1–5), 5 and 6 (56.1–9) as well as 7 and 8 (78.1–4).

### RNA sequencing

RNA was extracted from eye and wing imaginal discs using the Macherey-Nagel NucleoSpin RNA kit. The imaginal discs were disassociated by thorough vortexing in the RA1 buffer from the kit with β-mercaptoethanol. The DNase treatment of this kit was insufficient for qRT PCR analysis or RNAseq experiments, we therefore treated all samples with the Ambion (Life Technologies) RNA DNA-free DNase Treatment & Removal kit.

We tested four conditions (wild-type, *sev-wg*, *sev-wg* with *pygo*^*S123*^ and *sev-wg* with *bel*^*3R*.*382*.*1*^) with three biological replicas each: RNA was extracted from eye imaginal discs and RNAseq libraries for Illumnia HiSeq 2000 were prepared according manufacturer specifications. The machine was operated in single read mode at a read length of 100bp. The sequencing and data analysis was performed at the Genomics Platform of the Institute of Genetics and Genomics at the University of Geneva (Switzerland).

Fastq reads from the Illumina sequencer were checked for quality with FastQC [9] and mapped to the *Drosophila* reference genome dm3 using TopHat [10]. The biological QC was performed with picard (available on http://picard.sourceforge.net) and a table of counts established with HTseq [11]. The normalization and DE testing was performed with edgeR [12].

Statistical significance was tested by a paired, two-sided student’s T-test (p ≤ 0.05).

### Quantitative real-time polymerase chain reaction

RNA extraction was performed as for the RNAseq experiment. cDNA synthesis was carried out following standard procedures using the Transcriptor High Fidelity cDNA Synthesis kit from Roche. qRT-PCR reactions were performed in triplicates using MESA GREEN 2x PCR Master Mix for SYBR Green I Assays (4mM final MgCl_2_) and an ABI PRISM 7900 HT Sequence Detection System (SDS, Life Technologies) under the following conditions: 95°C for five minutes, followed by 40 cycles of 95°C for 15 seconds and 60°C for one minute, followed by 95°C for 15 seconds, 60°C for 15 seconds and 95°C for 15 seconds. Raw data was analyzed with SDS Relative Quantification Software version 2.2.3 (Life Technologies), generally using the automatic cycle threshold (Ct) setting for assigning baseline and threshold for Ct determination. To measure the Wnt output at the level of Wnt target gene expression, *wingful/Notum* (*wf*) was used as target gene and *Tbp*, *Tub84B* and *Act5C* were used as reference housekeeping genes. All primers were designed with CLC main workbench (CLC bio, QIAGEN) and ordered at Microsynth AG. Statistical significance was tested by a paired, two-sided student’s T-test (p ≤ 0.05). The primers used for qRT-PCR are listed in Table 2.

**Table 2:** List of all primers used for qRT-PCR.

Gene	Forward primer	Reverse primer
wf	AAATGGATTAAGCCACAGCAG	GGCAGCTGACTGAACGTG
grim	ATCGATGACCATGTCGGAGT	CGCAGAGCGTAGCAGAAGAT
Tbp	GCCAGATGCCGTGTGACAA	AGTCTCGCTGAAGAAGGTGTTGA
Tub	CGCGCATCATCCAAAAGC	GCCGACCATGTTTTGAATCTTAA
Act5C	GCCCATCTACGAGGGTTATGC	AATCGCGACCAGCCAGATC

## Results

### Bel is required for the Wg-induced small-eye phenotype

**Fig 1:**
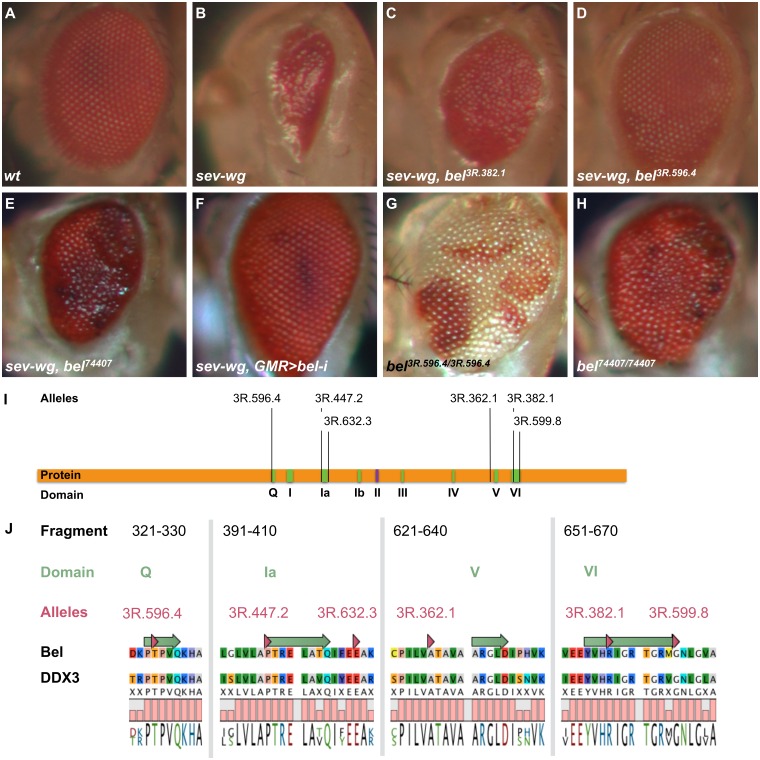
Eye phenotypes and structure-function analysis of *bel*. (A) Wild-type eyes show regularly structured ommatidia with interommatidial bristles. (B) The *sev-wg* transgene perturbs eye development and causes a small, rough eye phenotype. (C-E) Different *bel* alleles can suppress this phenotype: alleles (C) 3R.382.1 and (D) 3R.596.4 represent mis-sense mutations. (E) A P-element induced null allele and (F) RNAi knock-down of *bel* also suppress the *sev-wg* phenotype. (G) In a wild-type (without *sev-wg*) the mis-sense mutation 3R.596.4 leads to larger ommatidia and patterning defects. (H) A similar phenotype can be observed with the null allele of *bel*. (I) In a *sev-wg* suppressor screen we isolated five recessive and one dominant *bel* suppressor alleles, all mutations reside in conserved DEAD-box RNA helicase motifs or in close vicinity. (J) When Bel is compared to human DDX3X, all mutations are located in conserved regions.

In a forward genetic screen for suppressors of the *sev-wg* phenotype (caused by the ectopic expression of *wg* driven by *sevenless* (*sev*) enhancer elements)[13–16], we recovered five recessive and one dominant suppressor allele of *bel* (FH Jenny, M Hediger Niessen *et al*., manuscript in preparation). All of them were able to revert the glossy and small eyes caused by the *sev-wg* transgene to larger structures and partially restored ommatidial patterning; however, the lack of interommatidial bristles, a characteristic aspect of the *sev-wg* phenotype, was not rescued (Fig 1A–1D). A P-element induced *bel* null allele also suppressed the *sev-wg* phenotype, but not as effectively as our EMS alleles and it additionally caused the appearance of dark spots in the eye (Fig 1E). The suppression can also be observed using GMR-driven (glass multimer reporter) *bel* RNAi expression (Fig 1F). In the absence of the *sev-wg* transgene, homozygous *bel* mutant clones show eye patterning defects and occasional enlarged ommatidia (Fig 1G and 1H). Hence, alleles of *bel* are able to suppress a Wg-induced phenotype and cause eye patterning defects in a wild-type background.

### The suppressor mutations in *bel* map to the helicase motifs

To obtain structure/function information we sequenced our *bel* alleles (Table 3). Four of the six mutations reside within the highly conserved DEAD-box helicase motifs Q, Ia and VI, and the other two are located in very close proximity to motifs Ia and V, in regions which are conserved between human and fly homologues (Fig 1I and 1J). This suggests that the consequences of the ectopic Wg signaling phenotype depend on the helicase function of Bel; without this activity, the *sev-wg* phenotype is strongly suppressed.

**Table 3:** List of all *bel* alleles from the screen.

Allele	D	GP	NX	PP	AX
bel[3R.596.4]	recessive	3R:4,483,028	G to A	318	T to I
bel[3R.447.1]	dominant	3R:4,483,229	C to T	385	P to L
bel[3R.632.3]	recessive	3R:4,483,261	G to A	396	E to K
bel[3R.362.1]	recessive	3R:4,483,915	C to T	614	A to T
bel[3R.382.1]	recessive	3R:4,484,008	G to A	645	R to C
bel[3R.599.8]	recessive	3R:4,484,032	G to A	653	G to S

All genomic positions (GP) are based on the *Drosophila* genome release 5.22 and the protein positions (PP) are based on sequence Q9VHP0. Nucleotide exchanges (NX) and amino acid exchanges (AX) are also listed as well as the dominance behavior of the alleles (D).

**Fig 2:**
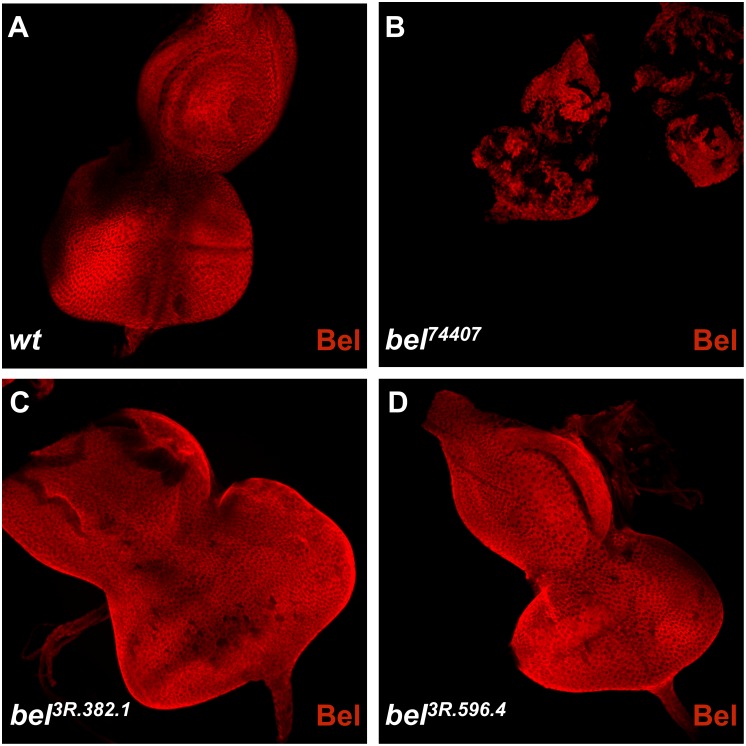
Immunohistochemical analyses of eye imaginal discs confirming that the mis-sense alleles encode stable Bel proteins and cause a reduction of cell death. (A) The wild-type Bel protein is localized in the cytosol in eye imaginal disc cells and it is uniformly distributed in the tissue; less intensely stained spots arise from the folding of the discs. (B) In clones homozygous for the *bel* null-allele, the staining is lost, which indicates a complete loss of Bel and confirms the specificity of the antibody used. Larvae carrying the null-allele grow slower; therefore the disc is smaller than wild-type discs and discs from other *bel* mutants. (C) In *bel*^*3R*.*382*.*1*^ the protein staining is similar to wild-type, suggesting a correct folding of the protein. (D) The same is the case for *bel*^*3R*.*596*.*4*^.

We next tested whether these missense mutations affect protein stability, and performed antibody staining against the products of the *bel*^*3R*.*382*.*1*^ and *bel*^*3R*.*596*.*4*^ alleles. The polyclonal antibody is directed against antigens in the N-terminal region (amino acids 1–230, reference) and recognizes both the wild-type as well as the mutant Bel proteins (Fig 2A, 2C and 2D). In eye disc clones homozygous for a *bel* null-allele, the Bel protein was absent and no staining could be observed (Fig 2B). The stability of the products of *bel*^*3R*.*382*.*1*^ and *bel*^*3R*.*596*.*4*^ alleles was not affected.

### Bel is not required for endogenous Wg signaling

**Fig 3:**
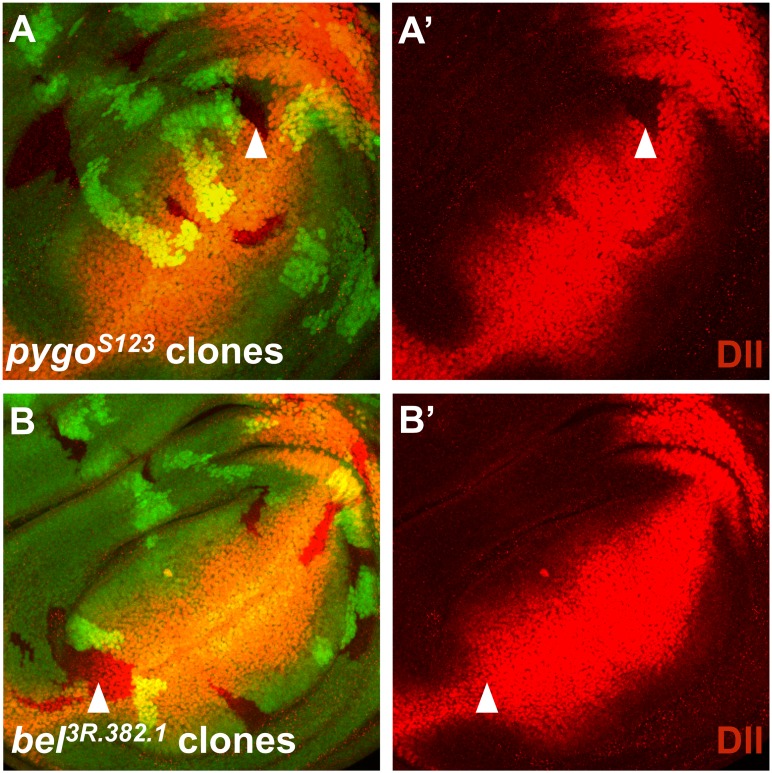
(A) Through heat-shock induced mitotic recombination, homozygous mutant clones were generated in wing imaginal discs and this allows us to study specific protein expression patterns of known Wg target genes. In the GFP channel black spots indicate homozygous mutant clones, bright green spots mark homozygous wild-type clones (twin spots) and weakly green cells comprise the heterozygous tissue. In clones homozygous mutant for *pygo*, a clear reduction in Dll expression can be seen. (B) In contrast, *bel* homozygous mutant clones do not exhibit altered Dll expression.

**Fig 4:**
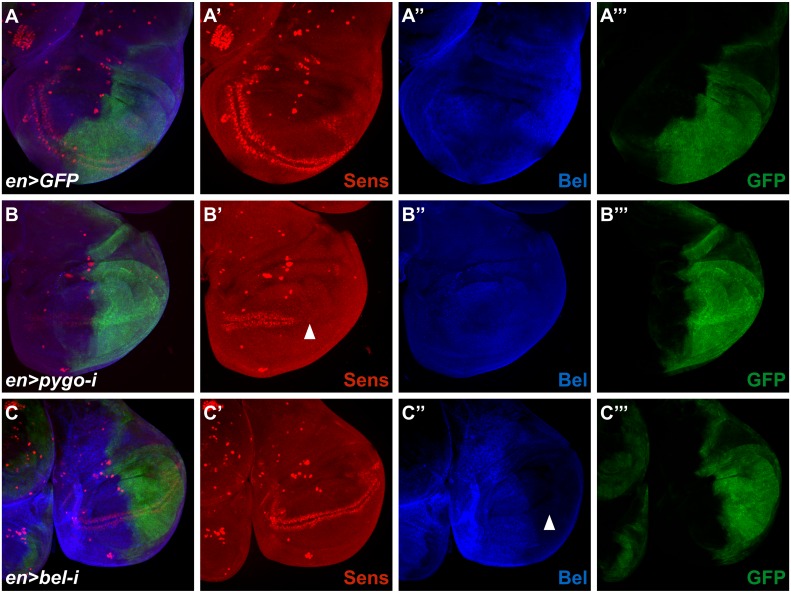
Loss of *bel* function does not affect Wg target gene expression. (A) Over-expression of a neutral GFP transgene in the posterior compartment using an *en* driver did not affect Sens distribution. (B) If the known Wg signaling component Pygo was knocked-down by RNAi the Sens staining in the posterior half of the disc was lost. (C) The knock-down of Bel did not show any reduction in Sens staining in P compartment cells.

Since we observed that mutations in *bel* can partially suppress the phenotype caused by ectopic Wg signaling, we tested whether Bel is required for the expression of Wg target genes. In the wing imaginal disc, established Wg targets are *senseless* (*sens*) and *Distalless* (*Dll*). In clones lacking *bel* function, neither the expression pattern, nor the protein levels of Sens or Dll were affected (Fig 3A and 3B). We repeated this experiment with *bel* RNAi, driven by regulatory regions of the *engrailed* (*en*) gene. While a severe reduction of Bel protein levels was observed, no effect on Sens expression was seen in the P compartment of wing discs (Fig 4A–4C). Adult animals with *en* driven *bel* RNAi did not exhibit any wing phenotype (data not shown).

**Fig 5:**
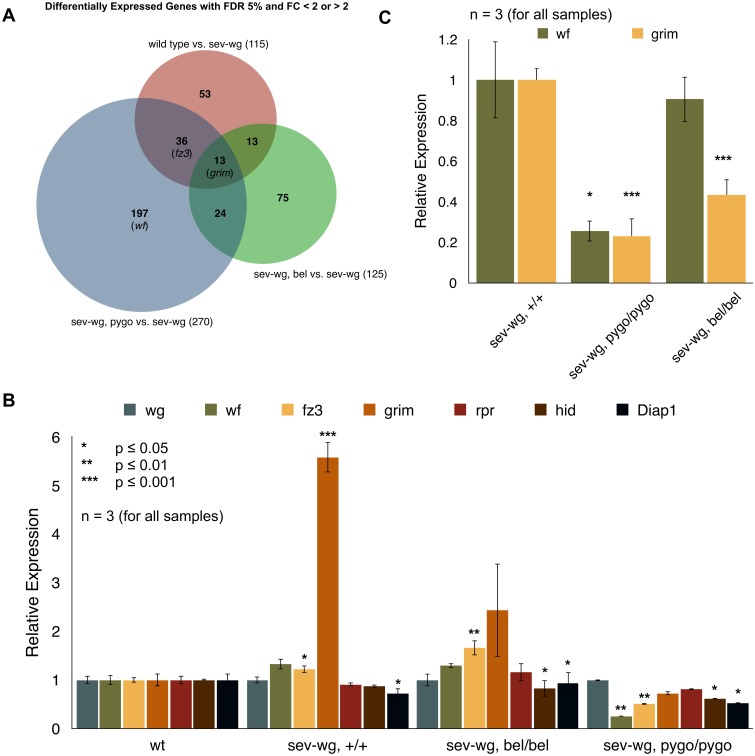
Transcriptional effects caused by *bel* mutations. (A) In an RNAseq experiment wild-type, *sev-wg bel* and *sev-wg pygo* imaginal discs were analyzed and compared to each other and to *sev-wg* discs. Several genes were differentially expressed with an FDR (false discovery rate) of 5% and FC (fold change) < 2 or > 2. (B) We compared expression levels of Wg target genes (*wf*, *fz3*) and apoptosis effectors (*grim*, *reaper* (*rpr*), *head involution defective* (*hid*) and *Death-associated inhibitor of apoptosis 1* (*Diap1*)) in the various genotypes. To account for potential differences in *wg* expression levels, we normalized all gene expressions these to these. To better compare the relative expression levels of the selected genes, an additional normalization was carried out, such that their expression levels in the wild-type genotype was set to 1. *grim* is up-regulated (approximately 5-fold) as well as *fz3*, *wf* shows a tendency of up-regulation (p<0.07) in the sev-wg genotype. While mutations in *bel* in the *sev-wg* background reduces *grim* expression, it does not appear to affect the *wg* target gene *fz3*. Mutated *pygo* in the *sev-wg* background reverts *grim* levels to wild-type and even further reduces *wg* target gene expression (*wf* and *fz3*). Transcript levels of other apoptosis genes, like *Diap1*, *hid* and *rpr* are not up-regulated by the *sev-wg* transgene (Diap1 shows a slight down-regulation). (C) We confirmed the RNAseq results by qRT PCR: the expression of the known Wg target gene *wf* and of the apoptosis pathway gene *grim*, which is known to be up-regulated by the *sev-wg* transgene. In eye discs homozygous mutant for *pygo* expression of the Wg target gene *wf* is reduced. This is not the case in homozygous mutant *bel* discs. However, mutations in both *pygo* or *bel* reduce *grim* expression.

We next examined the transcriptome of *sev-wg* eye imaginal discs that additionally were either wild-type or mutant for *bel* by RNAseq (S1 Table). As a comparison, we also sequenced the transcriptome of *sev-wg* discs that were either wild-type or mutant for *pygopus* (*pygo*, encoding a nuclear component required for transcriptional Wg outputs). To investigate the effect of the *sev-wg* transgene we also compared wild-type versus *sev-wg* eye discs. This comparison yielded 115 differentially expressed genes; *sev-wg pygo* vs. *sev-wg* yielded 270 and *sev-wg bel* vs. *sev-wg* yielded 125 differentially expressed genes. As illustrated in Fig 5A there is some overlap between the data sets. All three comparisons overlap in 13 differentially expressed genes, including the apoptosis gene *grim* (other apoptosis effector genes are unaffected, Figs 5B and S1). *sev-wg* causes a ca. 5-fold increase of *grim* expression (compared to wild-type discs), which is reverted to normal levels by *pygo* as well as by *bel* alleles. In contrast, the *sev-wg* induced *wf* and *fz3* levels are only reverted by the *pygo* genotype but not by the *bel* genotype (Fig 5B).

These experiments suggest that mutations in *bel* suppress the *sev-wg* gain-of-Wg signaling phenotype *downstream* of the canonical pathway (i.e. not altering Wg target gene expression), in contrast to the *pygo* mutation, which in addition to the phenotypic suppression also affects Wg target gene expression.

We confirmed these results using qRT PCR measuring transcript levels of *wf* and *grim* in a *sev-wg* background: while the *pygo* mutation is able to reduce *wf* expression in eye imaginal discs, *bel* mutations do not show a reduction of *wf* transcripts. However, *pygo* as well as *bel* alleles reduce the levels of *grim* transcripts (Fig 5C).

**Fig 6:**
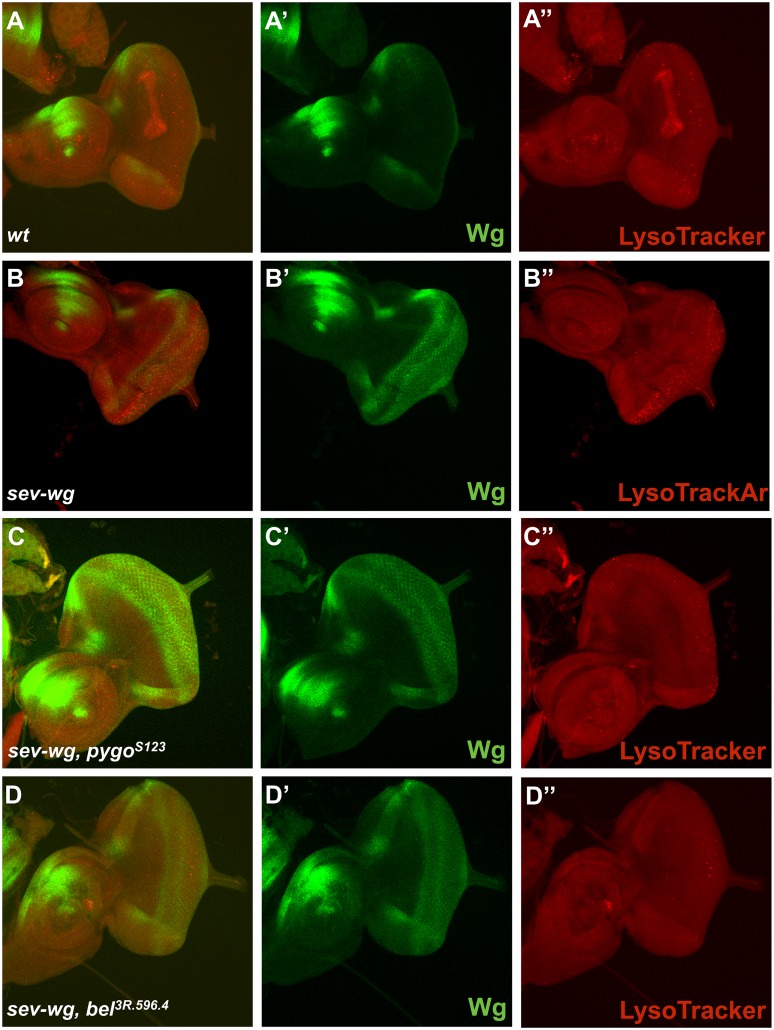
(A) LysoTracker is a highly specific probe for acidic organelles. In dying cells, the number of acidic lysosomes is increased, and hence LysoTracker can be used as a marker for cell death. Wild-type eye imaginal discs exhibit a moderate number of dying cells and (B) this number is strongly increased in *sev-wg* discs. (C and D) Homozygous mutant *pygo* and *bel* eye discs have a lower number of dying cells, similar to wild-type.

LysoTracker is an acidotropic dye that stains cells undergoing autophagy and apoptosis [17,18]. In eye imaginal discs LysoTracker staining shows that *sev-wg* induces cell death; in a background mutant for *pygo* or *bel* cell death is reduced to wild-type levels (Fig 6A–6D). These results suggest an interesting explanation for the suppression of the *sev-wg* phenotype in *Drosophila* eyes: Bel acts downstream of canonical Wg signaling but upstream of Grim-induced apoptosis, providing an explanation of why *bel* alleles suppress the eye phenotype yet fail to exhibit an effect on direct Wg target genes in wing and eye imaginal discs.

## Discussion

Our findings indicate that Bel plays a role in the suppression of the phenotype caused by ectopic Wg signaling in *Drosophila* eye imaginal discs. EMS derived alleles, a functional null allele (P-element induced) and *bel* knock down by RNAi, were all able to suppress the *sev-wg* phenotype. The *bel* alleles also showed an eye phenotype in a neutral background: these eyes are smaller and mis-structured, suggesting that Bel also plays a role in normal eye development.

We tested the role of *bel* in endogenous Wg signaling in eye and wing progenitor tissues. In wing discs, the protein expression of two known Wg target genes, Sens and Dll, was unaffected in homozygous mutant *bel* clones or in compartments where *bel* was knocked down. Measuring expression levels of the Wg target gene *wf* by qRT PCR and RNAseq in eye imaginal discs confirmed that Wg target gene expression does not depend on Bel. However, the *sev-wg* induced gene *grim* showed reduced transcript levels in *bel* mutants. Mutations in *pygo*, a bona-fide Wg pathway component, reduced both Wg target genes (*wf* and *fz3*) and *grim* expression. These findings suggest that Bel’s role lies downstream of the canonical Wg signaling cascade and upstream of *grim* expression. Our data is in contradiction with the recent report that the mammalian Bel homolog DDX3 is involved in the upstream Wnt signal cascade, acting as a regulatory subunit of CK1-ε, to promote the phosphorylation of Dsh [7]. This study attributed the Wnt/Wg signaling function to DDX3’s C-terminus (amino acids 456–662) and suggested it was independent of the helicase activity. The alleles we obtained from our EMS screen reveal a different picture. All mutations precisely map to conserved DEAD-box helicase motifs. This striking bias in localization of randomly induced mutations suggests that in *Drosophila* the helicase function plays a key role. Additionally, our analysis with the Bel specific antibody indicates that the mis-sense alleles are expressed at normal levels and thus should not affect a potential scaffold function of Bel.

The finding that Bel does not have a role in Wg signaling is not entirely unexpected in light of the mechanism proposed by Cruciat *et al*., who suggest that the relevant interaction partner is CK1-ε. In *Drosophila*, the role of CK1-ε in Wg signaling is uncertain. CK1-ε is implicated in non-canonical Wg signaling [19]. When overexpressed in cultured *Drosophila* cells *disc overgrown* (*dco*, encoding the *Drosophila* CK1-ε homolog) may affect canonical signaling. However, *in vivo* one study suggests that *sens* expression is reduced in *dco* clones [20], another study with the null-allele *dcoLE88* failed to find evidence for a role for Dco in the Wg pathway [21]. Further confusion is added by the finding that Bel was ascribed a negative role in Wg transduction based on the results of a genome-wide RNAi screen in *Drosophila* S2 cells [22].

Regardless of the role of DCO, our results show that in *Drosophila* Bel does not act as a core Wnt signaling component, however it is involved in the suppression of cell death induced by ectopic Wg signaling, downstream of the canonical pathway.

## Supporting Information

S1 Table Results of the examination of the transcriptome of *sev-wg* eye imaginal discs that additionally were either wild-type or mutant for *bel* by RNAseq. As a comparison, the transcriptome of *sev-wg* discs that were either fully wild-type or mutant for *pygopus* (*pygo*, encoding a nuclear component required for transcriptional Wg outputs).
Submission code: SUB1990968
STUDY: PRJNA347420 (Accession Number SRP091025)
(XLSX)

S1 Fig We compared expression levels of Wg target genes (wf, fz3) and apoptosis effectors (grim, reaper (rpr), head involution defective (hid) and Death-associated inhibitor of apoptosis 1 (Diap1)) in the various genotypes. To account for potential differences in wg expression levels, we normalized these. To better compare the relative expression levels of the selected genes, an additional normalization was carried out, such that their expression levels in the sev-wg genotype was set to 1. grim is down-regulated in all conditions, fz3 and wf only in wild type (wf p-value < 0.07) and sev-wg pygo. Transcript levels of other apoptosis genes, like Diap1, hid and rpr remain unchanged in most conditions (except Diap1 and hid in sev-wg, pygo eye discs and an increase of Diap1 expression in wild-type eye discs).
(PDF)
