# Supplementary figures and images for: Drosophila DDX3/Belle Exerts Its Function Outside of the Wnt/Wingless Signaling Pathway
Source: PLoS One. 2016 Dec 28;11(12):e0166862. doi: 10.1371/journal.pone.0166862 (PMC5193393; doi:10.1371/journal.pone.0166862)

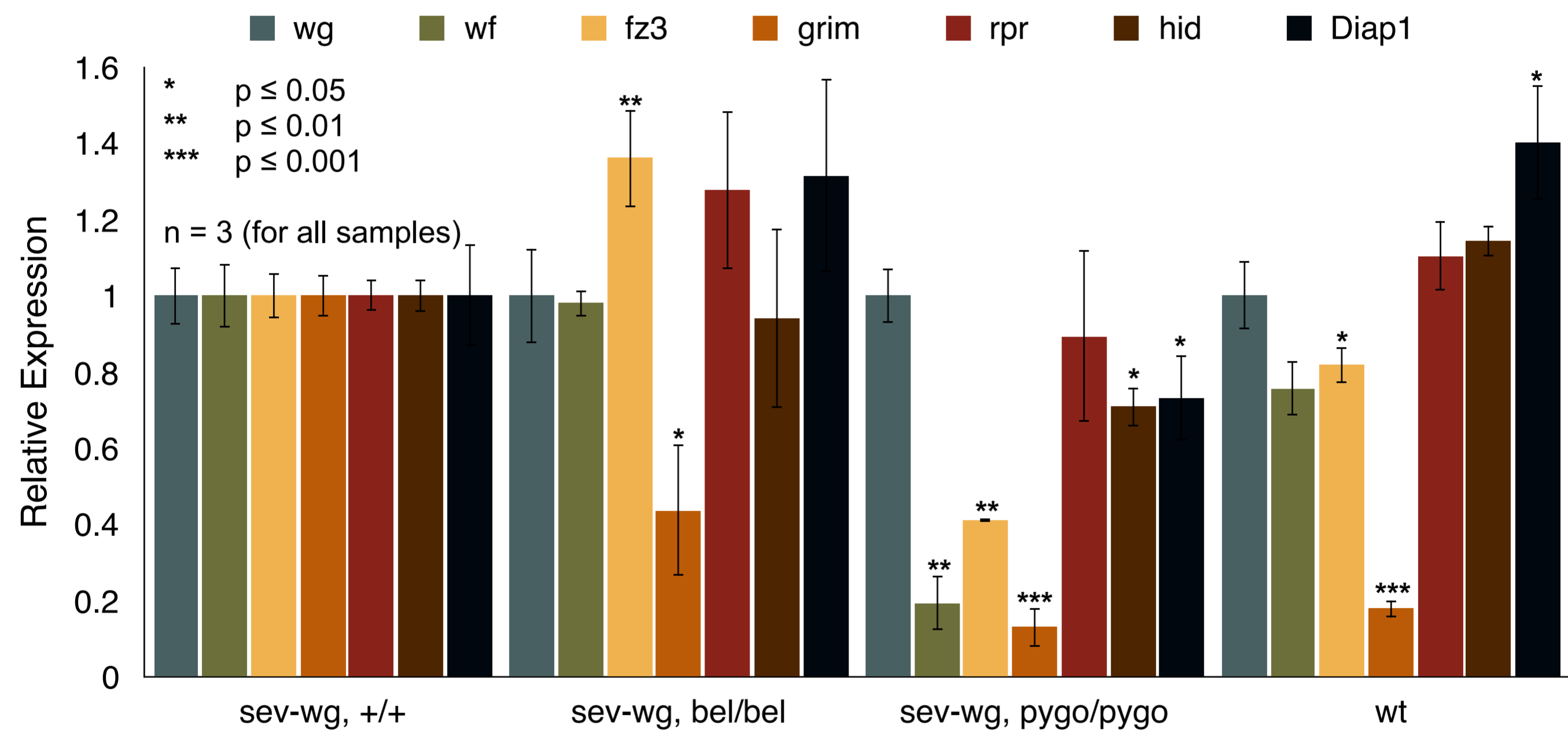

Supplement: S1 Fig — To account for potential differences in wg expression levels, we normalized these. To better compare the relative expression levels of the selected genes, an additional normalization was carried out, such that their expression levels in the sev-wg genotype was set to 1. grim is down-regulated in all conditions, fz3 and wf only in wild type (wf p-value < 0.07) and sev-wg pygo. Transcript levels of other apoptosis genes, like Diap1, hid and rpr remain unchanged in most conditions (except Diap1 and hid in sev-wg, pygo eye discs and an increase of Diap1 expression in wild-type eye discs). (PDF) [file pone.0166862.s002.pdf]
